# Inactivation of Poxviruses by Upper-Room UVC Light in a Simulated Hospital Room Environment

**DOI:** 10.1371/journal.pone.0003186

**Published:** 2008-09-10

**Authors:** James J. McDevitt, Donald K. Milton, Stephen N. Rudnick, Melvin W. First

**Affiliations:** 1 Department of Environmental Health, Harvard School of Public Health, Boston, Massachusetts, United States of America; 2 Department of Work Environment, University of Massachusetts Lowell, Lowell, Massachusetts, United States of America; University of Liverpool, United Kingdom

## Abstract

In the event of a smallpox outbreak due to bioterrorism, delays in vaccination programs may lead to significant secondary transmission. In the early phases of such an outbreak, transmission of smallpox will take place especially in locations where infected persons may congregate, such as hospital emergency rooms. Air disinfection using upper-room 254 nm (UVC) light can lower the airborne concentrations of infective viruses in the lower part of the room, and thereby control the spread of airborne infections among room occupants without exposing occupants to a significant amount of UVC. Using vaccinia virus aerosols as a surrogate for smallpox we report on the effectiveness of air disinfection, via upper-room UVC light, under simulated real world conditions including the effects of convection, mechanical mixing, temperature and relative humidity. In decay experiments, upper-room UVC fixtures used with mixing by a conventional ceiling fan produced decreases in airborne virus concentrations that would require additional ventilation of more than 87 air changes per hour. Under steady state conditions the effective air changes per hour associated with upper-room UVC ranged from 18 to 1000. The surprisingly high end of the observed range resulted from the extreme susceptibility of vaccinia virus to UVC at low relative humidity and use of 4 UVC fixtures in a small room with efficient air mixing. Increasing the number of UVC fixtures or mechanical ventilation rates resulted in greater fractional reduction in virus aerosol and UVC effectiveness was higher in winter compared to summer for each scenario tested. These data demonstrate that upper-room UVC has the potential to greatly reduce exposure to susceptible viral aerosols. The greater survival at baseline and greater UVC susceptibility of vaccinia under winter conditions suggest that while risk from an aerosol attack with smallpox would be greatest in winter, protective measures using UVC may also be most efficient at this time. These data may also be relevant to influenza, which also has improved aerosol survival at low RH and somewhat similar sensitivity to UVC.

## Introduction

Smallpox (*variola major*) is a high priority bioterrorist threat agent, according to the Centers for Disease Control and Prevention and Department of Homeland Security, which can be easily transmitted from person to person, result in high mortality rates, might cause public panic and social disruption, and require special action for public health preparedness (http://www.bt.cdc.gov/agent/agentlist-category.asp). Airborne spread via respiratory droplet nuclei has been identified as a potential contributing mode of transmission for smallpox[1], [2] and prevention of transmission by vaccination will likely be delayed until public health authorities become aware of the outbreak and initiate a vaccination program. In the early phases of such an outbreak, significant secondary transmission of smallpox will take place especially in locations where infected persons may congregate, such as hospital emergency rooms. Therefore, public health measures in addition to vaccination are needed.

Hospitals limit aerosol disease transmission in indoor spaces by reducing the concentration of airborne microorganisms through dilution ventilation. However, these measures are largely impractical beyond a limited number of respiratory isolation rooms due to the large amounts of air exchange needed to significantly reduce the threat of infection and are therefore costly in terms of heating and cooling these large amounts of air. The high ventilation rates required for respiratory isolation rooms are not routinely used in emergency departments and waiting areas. With air disinfection, costs are reduced since air does not have to be removed from occupied spaces to remove potential infectious agents. Disinfection using high-efficiency filtration to significantly reduce the threat of airborne infection can be effective but requires more powerful fans beyond what currently exist in the majority of public buildings and also require additional energy consumption. Air disinfection using upper-room 254 nm (UVC) light can lower the airborne concentrations of infective organisms in the lower part of the room, and thereby control the spread of airborne infections among room occupants without exposing occupants to a significant amount of UVC.[3]–[5] Upper-room UVC systems do not require modification to ventilation systems, are low maintenance, and relatively easy to install.[6], [7] The use of upper-room UVC is also economical. For example, the 25-watt lamps used as part of our study would cost just over $40 per year assuming an electrical cost of $0.20 per kilowatt-hour. Some hospitals currently employ upper-room UVC for this purpose in their emergency departments (e.g. Brigham and Women's Hospital, Boston, MA), but its effectiveness against viral aerosols is not well established.

Inactivation of microorganisms using UVC is often assumed to follow a first-order decay with a susceptibility parameter Z = ln(1/*f*) / D, where (*f* = organism fractional survival and D = UV dose, where dose is the product of UV fluence rate –expressed as power per cross sectional area—and exposure time, for example mJ/cm^2^. Using a one-pass UVC exposure chamber, however, we have shown that vaccinia virus (a surrogate for variola major) is susceptible to UVC and that the susceptibility varies as a function of dose and relative humidity (RH).[8] In these dose-response experiments the fluence rate and exposure time, and therefore, dose were carefully controlled. Thus, in each experiment, all viruses received the same dose and we determined susceptibility to UVC by varying dose over several experiments. In an actual room using upper-room UVC, the UVC fluence rate varies even within the upper-room, and the time spent in the upper-room varies from particle to particle. Therefore, the dose for each viral particle depends on the path that the particle travels. With perfect mixing, particle doses would be exponentially distributed. In the case of imperfect mixing, computational fluid dynamic (CFD) models should be capable of describing the more complex distribution of doses that would result. Then, using the pattern of UVC susceptibility we previously reported, it should be possible to estimate the net effectiveness of upper-room UVC. However, given the complexity of UVC susceptibility that we previously described combined with the complexity of CFD models, empirical data are needed. We report experiments designed to measure the effectiveness of upper-room UVC under simulated real world conditions including the effects of convection, mechanical mixing, temperature and relative humidity (RH).

## Results

### Decay

**Figure 1:**
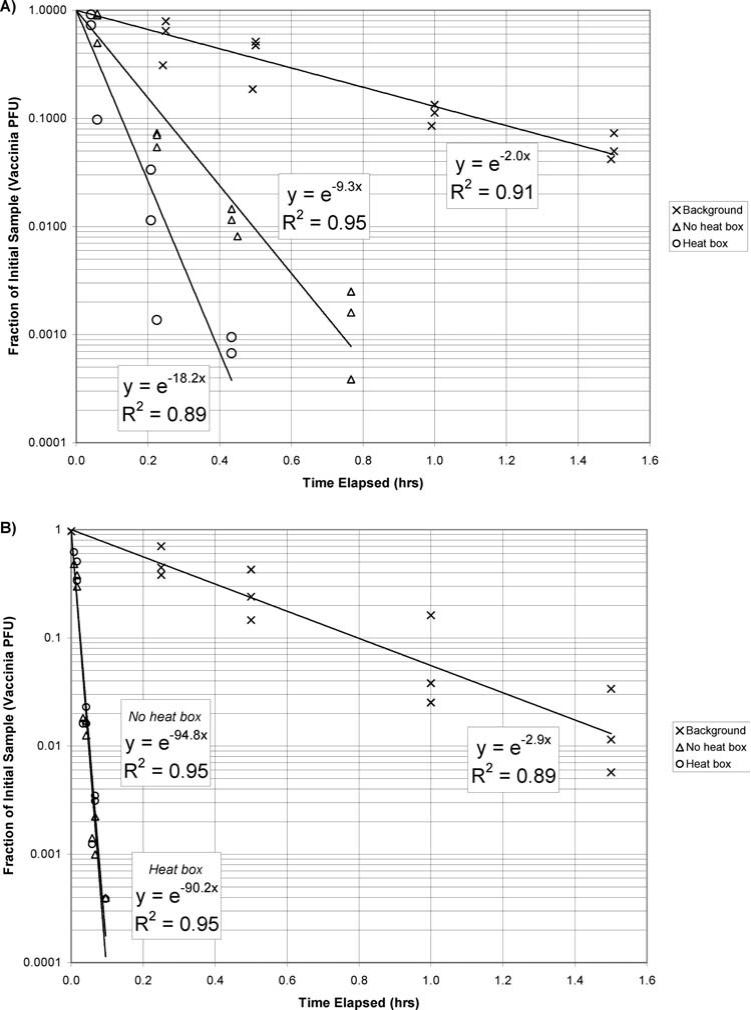
Background decay rates and decay rates for UVC light with and without heat boxes. a) ceiling fan is not operational; b) when ceiling fan is operational.

The environmental conditions within the chamber during decay experiment were maintained at 20±3°C and 50±10% RH. The results of chamber decay experiments performed with background decay, without heat boxes, and with heat boxes are shown either without the ceiling fan operating (Figure 1a) or with the ceiling fan operating (Figure 1b). The exponential regression model fits the data reasonably well. The rate constant shown in these equations can be interpreted as the effective air exchange rate for the chamber expressed in units of air changes per hour (ACH). Based on a model for a chamber in which the air is perfectly mixed, the effective air exchange rate is equal to the amount of virus-free dilution air that would be needed to provide the same reduction of virus concentration that was actually measured. The background decay rate reflects the decrease in infective viruses due to the exhaust airflow required to maintain negative pressure within the chamber, as well as any physical and non-UVC-related biological decay of the virus aerosol.

Virus reduction due to upper-room UVC is equal to the effective air exchange rate with the UVC turned on minus the effective air exchange rate with the UVC turned off (i.e. the background decay rate). This difference, which is usually referred to as equivalent air changes per hour (ACH_UVC_), is summarized in Table 1. Overall, the rate of reduction of vaccinia virus increased over the background as the amount of natural convection increased; mixing by the ceiling fan overwhelmed natural convection effects and markedly increased virus inactivation. When the ceiling fan was not operating, the ACH_UVC_ increased by 7 ACH above background when viruses were dispersed at 37°C (body temperature). When additional convective currents were added to the room by the addition of two heat boxes (equivalent to the heat generated by two people) the ACH_UVC_ increased by 16 ACH. When the ceiling fan was in operation the ACH_UVC_ increased to greater than 87 ACH and there was no discernable effect attributable to the heat boxes.

**Table 1:** Equivalent Air Changes per Hour Due to UVC (ACH_UVC_) for Virus Aerosol Decay Tests with and without ceiling fan and heat boxes.

	Ceiling Fan Operational	
	No	Yes
**Without Heat Boxes**	7	92
**With Heat Boxes**	16	87

### Steady State

**Figure 2:**
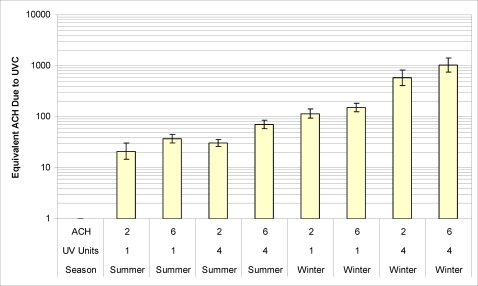
Equivalent air changes due to UVC under steady state conditions with either 2 or 6 ACH, 1 or 4 UVC fixtures, or winter and summer conditions.

The average concentration of vaccinia aerosols during steady state conditions with UVC off ranged from 1500 to 27000 pfu/m^3^. One experiment (summer conditions with 2 ACH and 4 UVC fixtures), in which the initial concentration before the UVC was turned on (1500 pfu/m^3^) was much lower than any of the other experiments was not used in our analysis, because the initial concentration was too low to accurately measure >85% reductions in concentration. The geometric mean vaccinia concentrations without UVC for the experiments used in the analysis were 3400 (95% confidence interval 2600 to 4300) pfu/m^3^ under summer conditions and 7800 (CI 5800 to 10000) pfu/m^3^ under winter conditions. With UVC on, the geometric mean concentrations were 570 (CI 430 to 770) pfu/m^3^ in the summer and 110 (CI 79 to 150) pfu/m^3^ in the winter experiments. The experiments under summer conditions showed stronger time trends in aerosol concentrations and greater variability between experimental replicates (i.e. steady state was difficult to achieve even without the UVC fixtures). The fraction of infectious virus remaining (ratio of the concentration of virus at steady state with upper-room UVC on to that measured under steady state conditions without UVC) is shown in Table 2 for the various combinations of tested conditions: two ventilation rates (2 and 6 ACH), numbers of UVC fixtures (1 or 4 fixtures) and seasonal conditions (summer and winter). Equivalent air changes due to UVC under steady state conditions for the various test conditions are shown in Figure 2. UVC achieved greater than 85% reduction in virus aerosol concentrations for all test conditions. Increasing the number of UVC fixtures from 1 to 4 resulted in greater fractional reduction in virus aerosol concentration at both 2 and 6 ACH. The fraction of virus surviving UVC treatment was lower under winter conditions compared to summer conditions. The additional effective air changes per hour due to UVC at each ventilation rate were 4 to 19 times greater during winter than summer.

**Table 2:** Effective Air Changes per Hour Associated with Upper-Room UVC for Steady State Virus Aerosols.

Condition	Replicate Trials (Air Samples)	Ventilation Rate, ACH	UVC Fixtures	Fraction of Infective Virus Remaining			ACH_UVC_		
				Estimate	95% Confidence Interval		Estimate	95% Confidence Interval	
Summer	2 (20)	2	1	0.087	0.062	0.120	18	15	30
	1 (12)	2	4	0.061	0.053	0.071	31	26	36
	3 (36)	6	1	0.14	0.120	0.160	38	31	46
	3 (36)	6	4	0.078	0.065	0.094	71	58	86
Winter	2 (24)	2	1	0.017	0.014	0.021	110	93	140
	2 (24)	2	4	0.003	0.002	0.005	580	410	830
	2 (24)	6	1	0.038	0.032	0.046	150	120	180
	2 (24)	6	4	0.006	0.004	0.008	1000	740	1400

## Discussion

These data show that in a ‘real world’ test setup, upper-room UVC is highly effective for reducing the concentration of vaccinia virus aerosols. We demonstrate through aerosol decay experiments that upper-room UVC fixtures used with mixing provided by a conventional ceiling fan and minimal general ventilation produced decreases in airborne virus concentrations that would require additional ventilation of more than 87 ACH. During steady-state experiments the combined effect of upper-room UVC and ventilation had a nonlinear impact on the fraction of remaining virus aerosol.[9] As a result, under winter conditions when vaccinia is most susceptible to UVC inactivation, the effective ACH due to upper-room UVC (ACH_UVC_) increased approximately five fold with increasing air exchange from ventilation. The equivalent ventilation achieved by UVC ranged from a low of 18 to 1000 ACH_UVC_, with winter equivalent ventilation rates consistently >100 ACH_UVC_.

The results from our decay experiments confirm the importance of vertical mixing cited for UVC effectiveness in model rooms.[3], [10] Vertical mixing is required to move organisms from the lower room into the upper-room where UVC intensity is the highest. Without vertical mixing, some viruses may get less UVC exposures or not get exposed at all and, as a result, UVC doses may be insufficient to cause deactivation.[9] During our decay experiments the ventilation system was operated so as to provide minimum negative pressure inside of the chamber to facilitate aerosol containment, while providing minimal mixing and dilution. As a result, when the heat boxes were not activated vertical mixing was primarily attributable to convection associated with the nebulizer diffuser which was heated to 37°C (approximately 17°C above the chamber temperature). The effective air exchange rate was a modest 7 ACH_UVC_ above the background. The effective ACH rate more than doubled (16 ACH_UVC_) when the heat given off by two people present in the room was simulated by the activation of the heat boxes. This value is consistent with the findings of Riley et el[11] for mycobacteria and are greater than what would be achieved by recommended dilution ventilation in hospital isolation rooms.[12] UVC and mixing using a ceiling fan together produced virus aerosol decay rates equivalent to 87 ACH_UVC_ and thus overwhelmed free convection effects. Similarly, First et al were able to show marked reduction in survival when comparing bacterial aerosol decay with and without ceiling fans in operation.[9]

Although these data are strong indicators that UVC would be an effective intervention, it has been recommended that tests of the efficacy of UVC against bioaerosols be based on steady-state measurements rather decay experiments.[10] We performed steady-state experiments under both summer and winter conditions. Consistent with early experiments on virus aerosol stability[13], [14], in the absence of UVC, vaccinia virus appeared to be more stable and higher aerosol concentrations were achieved with low RH (winter conditions) than with high RH (summer conditions). These experimental results also show that upper-room UVC is more effective when the relative humidity is low, even though mixing was reduced by operating the ceiling fans on a low, updraft, winter setting. These results are consistent with our bench-top experiments showing that vaccinia aerosols are more sensitive to UVC when relative humidity is low.[8]

Examining the fractional reduction of viral aerosol concentrations under various conditions clearly shows that upper-room UVC is capable of greatly reducing exposure. But, fraction reduction measurements do not easily translate into estimates of the actual level of risk achieved or facilitate decision making about how to best deploy upper-room UVC as part of a protection strategy. To estimate the level of risk with, for example, the Wells-Riley equation, we need to convert the fractional survival measurements into equivalent ventilation rates.[10], [15] This is easily done because at steady-state the ratio of total effective sanitary ventilation (Q*_UV_*+Q) to actual ventilation through air movement is equal to the ratio of virus concentration without UVC to the concentration with UVC (the inverse of the remaining fraction *f_ss_*, i.e. (*Q_UV_*+*Q*)*/Q* = *f_ss_* ^−1^), where Q_UV_ stands for the supply of virus free air due to UV (see Appendix S1) and Q is the ventilation rate with infective-virus-free air (m^3^/s).[9] If the fraction of infectious virus remaining were constant when the air exchange was tripled, then the total effective sanitary ventilation and effective ventilation due to UVC would also be tripled. However, in these data, when we tripled the air exchange rate from 2 to 6 ACH, the fraction of infectious virus remaining increased. This does not, however, imply that upper-room UVC gives less protection when ventilation is increased. It is true that while increased ventilation reduced the virus aerosol concentration it also reduced the average residence time of viral particles resulting in lower UVC doses to individual particles. But, the increase in *f* was not great enough to completely offset the more than additive effect of increased air exchanges. When we increased the air exchange rate from 2 to 6 ACH, a factor of 3, the effective ventilation due to UVC increased by a factor of 1.3 to 1.9. Thus, increased ventilation actually increased UVC fixtures effectiveness in terms of ACH_UV_ – the combination of ventilation and upper-room UVC is more than merely additive.

With one UVC fixture under summer conditions, when we increased *Q* by 4 ACH the effective ventilation from UVC increased by 20 ACH. In the winter with one fixture, when we increased the air exchange by 4 ACH, the effective ventilation from UVC increased by 40 ACH, and with 4 fixtures the effective ventilation increased by 420 ACH. The high UVC susceptibility of vaccinia when RH is low, i.e. the very small *f* observed under winter conditions, and the nonlinear interaction of UVC disinfection with ventilation produced extremely highly effective ventilation when the two were combined – ranging from >100 to 1000 ACH. In our previous bench top, dose-response studies of vaccinia virus, moderate UVC doses (3 J/m^2^) reduced vaccinia survival by a factor of >10,000 over natural biological decay.[8] For the present study we used an equation developed by Rudnick and First[16], that relates UVC fixture power output to *mean* fluence rate for the entire room, to estimate the *mean* UVC dose for the entire room assuming near perfect mixing, one fixture was in use, and a ten minute exposure time (i.e. 6 AC/hr). Under these conditions the UVC dose was estimated to be 17 J/m^2^. With four fixures in use the dose would be expected to be 4-times higher. Thus, the fraction of virus surviving, especially when they are most susceptible, would be expected to be quite low.

Studies by other researchers have made similar measures of UVC light effectiveness under steady-state conditions with bacterial aerosols. Bacteria such as *bacillus subtillus* and *serratia marcescens* have been used in full scale tests of upper-room UVC[4], [9] Bacteria are much more resistant to UVC light and have a correspondingly lower UVC susceptibility parameter, referred to as a Z-value. Riley and Kaufman noted decreased susceptibility to UVC for *Serratia Marcescens* exposed to UVC when RH exceeded 60% RH.[19] Ko et al noted a similar RH trends with *S. marcescens* and *Mycobacterium bovis* aerosols exposed to UVC.[20] Our previous studies of Vaccinia virus aerosol showed vaccinia virus susceptibility was highest when relative humidity was low.[8] Thus, the very high UVC susceptibility of vaccinia virus, especially when relative humidity is low [8], most likely accounts for the extremely high effective air changes per hour associated with UVC we found in comparison to studies[3] using bacterial aerosols. First et al. evaluated vaccinia virus in a full-scale chamber under steady-state conditions at 50% RH and reported similar results to our summer conditions.[9]

The additional effective ventilation due to upper-room UVC, even in the summer, ranged between 18 and 71 ACH, rates in excess of what is usually achieved in hospital rooms designed for airborne precautions (approximately 12 ACH) [12]. The additional effective ventilation achieved under winter conditions was phenomenal (110 with a single fixture to 1000 ACH with four fixtures). These data demonstrate that upper-room UVC has the potential to greatly reduce exposure to susceptible viral aerosols. The greater survival at baseline and greater UVC susceptibility of vaccinia under winter conditions suggest that while risk from an aerosol attack with smallpox would be greatest in winter, protective measures using UVC may also be most efficient at this time. These data may also be relevant to influenza, which also has improved aerosol survival at low RH. Given current concern about potential for a pandemic in the near future, and the potential that an important fraction of influenza transmission occurs via aerosols, further studies of UVC susceptibility and upper-room UVC effectiveness for influenza are warranted.

## Materials and Methods

### Experimental Chamber

**Figure 3:**
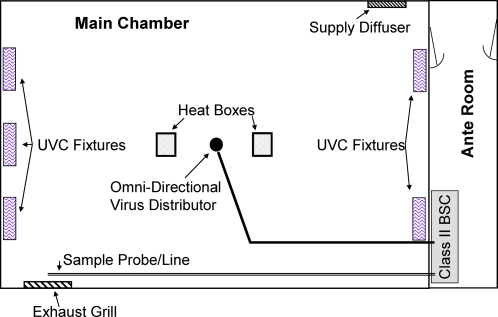
Schematic diagram of aerosol chamber and equipment. Note: for clarity ceiling fan is not shown, but is located in the center of the main chamber directly above the virus distributor. For decay and single fixture steady state experiments the fixture shown in the center of the wall on the left of the figure was used and for the four fixture steady state experiments the other four fixtures were used.

The testing chamber, ante room, aerosol generation, and sampling arrangements have been described previously and are shown in Figure 3.[9] Briefly, virus aerosols were delivered at 1.5 meters above the floor in the center of a climate controlled 4.60 m×2.97 m×3.05 m high room equipped with a ceiling fan and two black boxes containing 100-watt light bulbs (simulate body heat of two people). The boxes were located approximately one meter from the center of the room. UVC light was provided by combinations of 5 wall-mounted Hygeaire UVC fixtures (Model LIND24-EVO; Atlantic Ultraviolet Corp., Bay Shore, NY), each using one 25-W low pressure mercury discharge lamp with a UVC output of 5W. Fixtures were mounted 2.3 m from the floor and experiments with one fixture used a single fixture pointed down the middle of the long axis of the room, while experiments with four fixtures used two fixtures on each end of the long axis mounted one meter from the wall corners.

### Aerosol generation and sampling

Vaccinia virus stock, Western Reserve strain, was prepared to a concentration of 10^7^–10^8^ plaque forming units (PFU)/ml as reported previously[8]. Vaccinia stock solution was suspended in phosphate buffered saline (PBS) with 10% fetal bovine serum and 20 µl of Antifoam A (Sigma, St. Louis). Vaccinia virus aerosols were generated using a 6-jet Collison nebulizer (BGI Inc., Waltham, MA) operating at 138 kPa. The nebulizer was located in a class II biological safety cabinet (BSC) in the ante room and attached to a permanently installed pipe leading to the center of the test chamber. An omni-directional diffuser was attached to the end of the pipe at 1.5 meters above the floor. The pipe was heated to (37°C).

A port for aerosol sampling was located in front of the exhaust grill and was connected via a pipe to a valve located within the BSC in the control room. Air was drawn through a two-way valve into either a 37 mm gelatin filter (SKC, Inc.Eighty Four, PA) housed in a polyethylene cassette or through a bypass at 28.3 lpm. Bypass or filtered air was then directed through a high efficiency particulate air (HEPA) filter located before the high volume sampling pump. The bypass was used to clear the dead space in the sampling tube prior to sampling (60 sec) and when changing the filters. Filters were dissolved and vaccinia viruses were enumerated by plaque assay on confluent layers of Vero cells as previously described.[8]

### Decay experiments

Vaccinia was aerosolized for approximately 30 minutes to achieve sufficiently high concentrations of virus to allow detection after multiple logs of reduction. The generation was stopped and 5-minute samples were taken at 5 to 10 min intervals for up to 90 min. The aerosolization and sampling procedure was repeated with one UVC fixture on (Figure 3) alternating with no UVC fixture decay runs. Each experiment consisted of three pairs of runs with UVC on and off. Decay experiments were carried out without heat boxes or ceiling fan, with heat boxes, and with heat boxes and the ceiling fan.

### Steadystate

UVC inactivation of vaccinia virus was tested under steady state conditions while simulating indoor summer (20°C, 80% RH, ceiling fan directing air downwards) and indoor winter (20°C, 40% RH, ceiling fan directing air upwards) environmental conditions, with either 2 or 6 ACH ventilation rates, and either 1 or 4 UVC light fixtures (Figure 3). We assumed that 3 air changes were sufficient to establish a 95% chamber equilibration. Thus, virus suspension was nebulized for 30 minutes prior to sampling to achieve steady-state at 6 ACH and 1.5 hours for 2 ACH. Triplicate sequential samples were collected with the UV fixtures off followed by activation of the UVC fixtures and 3 air changes to allow equilibration. Then, triplicate sequential samples were collected with the UVC fixtures on. The fixtures were then turned off and the cycle of sampling with fixtures off was repeated. Each sample was assigned a time of collection as the midpoint of the sampling interval relative to the start of virus nebulization.

### Data Analysis

Decay experiment observations for the number of pfu/m^3^ for each sample were divide by the pfu/m^3^ in the initial sample collected after aerosolization was complete (collected approximately from t = 0 and t = 5 min) to obtain an estimate of the fraction of infectious virus remaining at each time point within an experiment. Each estimate of fraction remaining was assigned to the midpoint of the sampling interval. Thus, t = 2.5 min was assigned the preliminary value 1.0 for fraction remaining. An exponential decay curve was fit to these data using following equation:
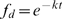
where *f_d_* is the fraction of infectious virus surviving and *k* is a rate (or decay) constant, and *t* is time. Each estimate of the fraction remaining was then adjusted by dividing all fractions remaining by the y-intercept of the regression. These adjusted fraction remaining estimates from each of the triplicate experiments with the same conditions were combined in a single regression to estimate the exponential decay constant (i.e. the effective air exchange rate). The equivalent air exchange rate due to UVC is the difference between the decay constant when UVC is in use and when it is not.[9]

For steady-state experiments, we computed pfu/m^3^ for each air sample. For each set of experimental conditions, we regressed ln(pfu/m^3^) on time, time squared, an indicator variable for operation of the UVC fixtures, an indicator for each experiment, and interactions of experiment indicators with the time variables. This allowed us to determine the effect of UVC controlled for experiment specific time trends in nebulizer output and variations in the virus aerosol concentrations achieved in each replicate experiment. The resulting coefficient for the indicator of UVC operation was the log of *f_ss_*, the ratio of steady state concentration of infectious virus with and without UVC, averaged over the replicate experiments. The ACH due to UVC was then computed as λ_U_ = λ_o_(1−*f*)/*f_ss_* where λ_o_ is the ACH due to ventilation (See Appendix S1 for derivation). Regression analyses and confidence limits for regression coefficients were computed using R statistical software (R-Project, Version 2.6.0) and summarized in Excel (Microsoft Corp, Redmond, WA).

## Supporting Information

Appendix S1 Derivation of Equivalent Air Exchange Rate Due to UVC. Derivation of equation used in data analysis.
(0.03 MB DOC)
