# Supplementary material for: Inactivation of Poxviruses by Upper-Room UVC Light in a Simulated Hospital Room Environment
Source: PLoS One. 2008 Sep 10;3(9):e3186. doi: 10.1371/journal.pone.0003186 (PMC2527528; doi:10.1371/journal.pone.0003186)
Supplement: Appendix S1 — Derivation of Equivalent Air Exchange Rate Due to UVC. Derivation of equation used in data analysis. (0.03 MB DOC) [file pone.0003186.s001.doc]

Appendix: Derivation of Equivalent Air Exchange Rate Due to UVC

Under steady-state conditions, dilution ventilation for the well-mixed airspace in a room can be modeled by the following equation:[[1]](#endnote-2)

$G=CQ$ (1)

where *G* is the generation rate of a contaminant such as infective vaccinia virus (pfu/s), *C* is infective virus concentration (pfu/m^3^), and *Q* is ventilation rate with infective-virus-free air (m^3^/s). If there are two sources of virus free air, we can partition Q into Q_1_ and Q_2_  such that Q = Q_1_ + Q_2_. Steady-state conditions will result in different concentrations depending on the amount of ventilation – but if the generation rate remains the same then,

$C_{1}Q_{1}=C_{2}(Q_{1}+Q_{2})$ (2)

If we let Q_2_ represent the supply of virus-free air due to UV (Q*_UV_*) and Q_1_ represent that supplied by ventilation (Q), and C and C_uv_ represent the concentrations at steady state without and with UVC, then

$f_{ss}=\frac{C_{uv}}{C}=\frac{Q}{Q+Q_{uv}}$ (3)

Where *f_ss_* is the ratio of stead-state concentrations with and without UVC. If we divide the numerator and denominator on the right by V, the volume of the room, then

$f_{ss}=\frac{C_{uv}}{C}=\frac{\lambda}{\lambda+\lambda_{uv}}$ (4)

where *λ*, the air exchange rate (s^-1^) due to ventilation and *λ_uv_* is the equivalent air exchange rate due to UVC. Then solving for *λ_uv_,* we have

$\lambda_{uv}=\frac{1-f_{ss}}{f_{ss}}\lambda$ (5)

1. Burgess, WA, Ellenbecker, MJ and Trietman, RD. Ventilation for control of the work environment, 2^nd^ edition, Hoboken NJ: John Wiley & Sons, 2004: 95. [↑](#endnote-ref-2)
